# Immersive Analytics: Theory and Research Agenda

**DOI:** 10.3389/frobt.2019.00082

**Published:** 2019-09-10

**Authors:** Richard Skarbez, Nicholas F. Polys, J. Todd Ogle, Chris North, Doug A. Bowman

**Affiliations:** Center for Human-Computer Interaction, Virginia Tech, Blacksburg, VA, United States

**Keywords:** immersive analytics, visual analytics, immersion, virtual reality, visualization, sensemaking, knowledge generation

## Abstract

Advances in a variety of computing fields, including “big data,” machine learning, visualization, and augmented/mixed/virtual reality, have combined to give rise to the emerging field of *immersive analytics*, which investigates how these new technologies support analysis and decision making. Thus far, we feel that immersive analytics research has been somewhat *ad hoc*, possibly owing to the fact that there is not yet an organizing framework for immersive analytics research. In this paper, we address this lack by proposing a definition for immersive analytics and identifying some general research areas and specific research questions that will be important for the development of this field. We also present three case studies that, while all being examples of what we would consider immersive analytics, present different challenges, and opportunities. These serve to demonstrate the breadth of immersive analytics and illustrate how the framework proposed in this paper applies to practical research.

## 1. Introduction

We are living and working in the era of “big data,” according to Kurose and Marzullo (2016). Information such as online activity, news media, health records, social media posts, geolocations, and networks of authors are all tracked, collected, aggregated, and stored. But it is not enough to have the data; the data must be analyzable to make it actionable. This paper explores ways that information visualization, machine learning, and virtual environments can come together to support analysis of big data. Specifically, we address the multiplicity of ways these fields combine to support *immersive analytics*.

There are two distinctly different—but complementary—approaches to big data analytics (Bertini and Lalanne, 2009). First, human analysts can sift through the data. Based on expertise, experience, and intuition, the best analysts can synthesize disparate information into cohesive hypotheses. Interactive visualization helps analysts view, organize, and synthesize the data (Van Wijk, 2005). But limitations in human capacity, plus the sheer volume of data, make human-only analysis intractable for many problems at scale. The second approach is to make use of machine intelligence, through data mining and machine learning algorithms, to forage for patterns and insights in huge datasets that would be overwhelming for human analysts. This approach has been very successful, but primarily for well-defined problems (Lazer et al., 2009). When it comes to sensemaking tasks requiring human intuition and pattern recognition (or a deep understanding of semantics), a combined approach is needed (Crouser and Chang, 2012; Counts et al., 2014). The varying ways these approaches can be combined are discussed at greater length in section 3 and in **Figure 2**.

**Figure 1:**
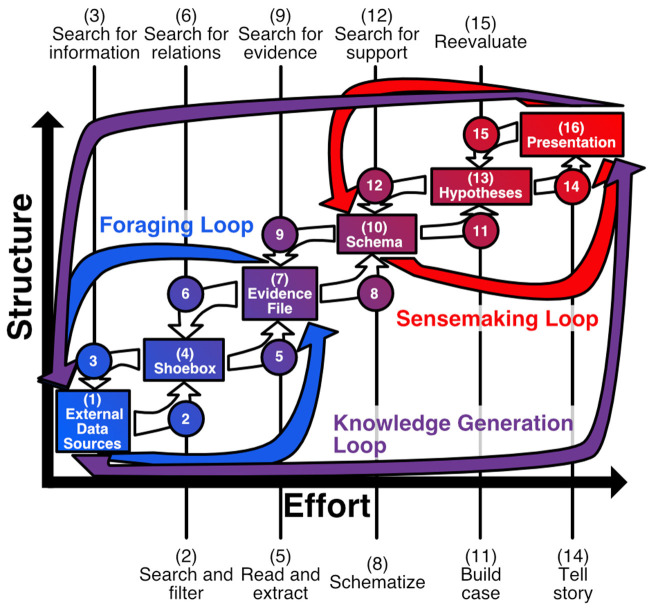
Knowledge generation loop (inspired by Pirolli and Card, 2005). The knowledge generation process can be organized into two main subprocesses: a foraging loop that seeks and organizes evidence, and a *sensemaking* loop that develops a mental model that fits the evidence.

The knowledge generation process of Pirolli and Card (2005) conceptually models how raw data is converted into a theory by human analysts via a series of analytical steps. These steps form two bidirectional loops: a foraging loop for uncovering new data, and a sensemaking loop for synthesizing that information into structured hypotheses (see Figure 1). By iterating the loops, analysts “incrementally formalize” their understanding of the data (Shipman et al., 1995). A goal of many visual analytics applications is to support user sensemaking processes with as unobtrusive a design as possible. Note that Pirolli and Card use the term “sense making” to refer to both the overall process, as well as just the latter part of the process. To avoid confusion, we use the term *knowledge generation* to refer to the overall process, and reserve the term sensemaking for the subprocess.

Recent trends have turned toward “human in the loop” analytics, a paradigm by which analysts provide feedback to analytical models in order to steer the computational path of those models (Andrews et al., 2010; Endert et al., 2012a, 2014). Leman et al. (2013) proposed a more user-centered variant, “human *is* the loop,” in which the focus lies on models assisting users in context of their sensemaking process, rather than users assisting models. While the foraging portion of the sensemaking process has gained much support from computational algorithms, the synthesis portion of the process has remained largely the domain of human abductive reasoning.

Interactive visual analytics recognizes the limitations of these two approaches, and proposes a hybrid approach in which human analysts are assisted by machine intelligence (Cook and Thomas, 2005; Keim et al., 2008). However, it is an open question as to what form this human-machine collaboration is to take. Immersive analytics situates this collaboration in an immersive virtual reality (VR) or augmented reality (AR) context.

With the advent of low-cost, high-quality VR/AR systems, many researchers are thinking of using such systems for visual analytics (dubbed *immersive analytics* by Chandler et al., 2015). However, what is usually meant by “immersive analytics” is the visualization of abstract datasets in an immersive 3D environment, as in Bowman et al. (2003), Henry and Polys (2010), Hossain et al. (2012), Bacim et al. (2013), Radics et al. (2015), Kwon et al. (2016), and Cordeil et al. (2017a). In this sort of immersive analytics, visualizations are designed in advance, and users primarily have the goal of foraging: examining the dataset visually to find items, clusters, or trends of interest.

In this paper, we suggest a definition of immersive analytics that goes beyond traditional visualization and is more consistent with the approaches used in visual analytics. Based on this definition, we propose a research agenda for this emerging field. Finally, we illustrate the challenges and opportunities of immersive analytics with three case studies.

## 2. What Is Immersive Analytics?

In Chandler et al.'s foundational paper, they describe immersive analytics as follows:

“Immersive Analytics investigates how new interaction and display technologies can be used to support analytical reasoning and decision making. The aim is to provide multi-sensory interfaces for analytics approaches that support collaboration and allow users to immerse themselves in their data. Immersive Analytics builds on technologies such as large touch surfaces, immersive virtual and augmented reality environments, haptic and audio displays, and modern fabrication techniques (Chandler et al., 2015).”

This provides a clear view of the field of study and its major components, but is somewhat resistant to further analysis. In the interest of informing our research agenda for immersive analytics, we propose an alternative definition. Specifically, we define *immersive analytics* as the science of analytical reasoning facilitated by immersive human-computer interfaces. In this, we follow Cook and Thomas (2005), who defined visual analytics as “the science of analytical reasoning facilitated by interactive visual interfaces.” That said, what is meant by the “science of analytical reasoning?” And which human-computer interfaces are considered “immersive?”

We first consider *analytical reasoning*. This term is quite broad; one can easily imagine analytical reasoning that consists of pure thought, without the aid of any technology at all. For our purposes, we will restrict our consideration to *computer-aided* analytical reasoning. Regarding analytical reasoning itself, we consider it to be practically equivalent to knowledge generation, as formulated by Pirolli and Card (2005) and addressed in section 1. We therefore propose that computer-aided analytical reasoning involves computer assistance in either or both of the foraging and sensemaking processes.

We now turn our attention to immersive human-computer interfaces. We follow Slater and others in defining *immersion* as an objective characteristic of a computer system, and specifically, as the set of valid actions supported by a the system, as seen in Slater (1999) and further explicated in Skarbez et al. (2017). Here, a *valid action* is defined as an any action a user can take that causes in a change to the internal state of the system (an *effective* valid action) or to the presentation of the system state (a *sensorimotor* valid action).

From here, one can see that when we refer to immersive human-computer interfaces, what we really mean is *more* immersive human-computer interfaces, as all computing systems that enable user input and output have some degree of immersion. There are many ways in which a system can be more immersive than such a typical workstation, as discussed in Bowman and McMahan (2007). Some specific technologies that would make a system more immersive include:

- Support for additional channels of sensory output
  − Haptic display
  − Olfactory display
  − Other sensory displays
- Capability to render existing channels of sensory output with greater fidelity (as in Polys et al., 2016)
  − “Large” visual display (substantially more than 30° FoV)
  − “Surrounding” visual display (i.e., head-worn displays, CAVE-like (Cruz-Neira et al., 1993) displays)
  − Higher-resolution visual display (e.g., “retina” resolution)
  − Stereoscopic visual display
  − Spatialized sound display
- Support for additional types or channels of user input
  − Voice input
  − Touch input
  − Physiological sensing
  − Tracking
    ° Head tracking
    ° Hand tracking
    ° Full-body tracking
    ° Eye tracking
- Support for more natural interaction techniques
  − Body-based navigation (i.e., physical head turning, walking, crouching)
  − Natural manipulation (e.g., directly touching/grasping/moving data with the hand)
  − Conversational speech interfaces
  − Gestural interfaces

Having now explicated both the “immersive” and “analytics” parts of immersive analytics, we now revisit our definitions:

- *Immersive analytics* is the science of analytical reasoning facilitated by immersive human-computer interfaces.
- By *analytical reasoning*, we specifically refer to computer-aided analytical reasoning as a partner with the human; that is, a process of foraging and sensemaking where part or all of the foraging and/or sensemaking processes are performed in cooperation with a computer.
- By *immersive human-computer interfaces*, we specifically mean those interfaces which enable a user to interact with a system using additional or more-immersive displays and user interface techniques.

## 3. Research Agenda

In section 1, we commented on the technological trends that are combining to give rise to immersive analytics at this point in time. However, it can be reasonably argued that the fact that immersive analytics is now practical does not automatically make it a good idea or a productive research direction. The onus falls on those of us in this field to justify the utility of immersive analytics to analysts with problems to solve. To do otherwise risks putting the cart before the horse.

There are some good reasons to believe that immersive analytics can indeed offer significant benefits to data analysts, beyond those offered by more traditional analytics applications. Bowman and McMahan (2007) identified several potential benefits of increased immersion, including increased spatial understanding due to integration of more depth cues, decreased information clutter due to increased effective display size, peripheral awareness, and information bandwidth. The results of the study performed by Gruchalla (2004) provide evidence in support of these hypotheses. In his study, sixteen participants performed a complicated spatial task–oil well path planning–using both a desktop workstation with a stereoscopic display and a CAVE-like (Cruz-Neira et al., 1993) immersive virtual environment. Fifteen of the sixteen participants performed the task faster in the more-immersive condition; this was accompanied by a statistically significant increase in correct solutions identified in the more-immersive condition. We have also suggested (section 4.2) that immersive analytics applications can provide extensive “space to think,” mimicking the ways in which analysts naturally use physical space to organize their analysis. Evidence indicates that performance on analytics tasks improves both quantitatively and qualitatively when physically larger displays are used (Andrews et al., 2010). This extends the results from Ball et al. (2007), where the authors observed several benefits of using physical locomotion (such as walking toward or away from the screen) rather than virtual locomotion (zooming using abstract user interface techniques) with large displays. Finally, we hypothesize that immersive analytics systems can take advantage of rich semantic interaction (Endert et al., 2012a) to infer analyst intent and help guide intelligent algorithms that support the analysis. While these results do not prove that immersive analytics is the preferred solution for every analysis application, they at least suggest that it is worthy of further investigation.

To aid in this investigation, this section outlines a research agenda for immersive analytics, based on the definition and discussion in section 2. We generalize and describe five areas of research:

- Combining Human and Computer Intelligence
- The Utility of Immersion
- Designing Immersive Analytics Systems
- Facilitating Collaboration through Immersion
- Changing the Process of Analysis with Immersion.

### 3.1. Combining Human and Computer Intelligence

In section 1, we suggested that a core premise of visual (and immersive) analytics is that a combination of human and machine intelligence may be able to achieve insights that could not be reached by either one alone. Arguably, then, the central question for immersive analytics is: *How can machine intelligence and human intelligence be most productively combined to address analytics tasks?*

**Figure 2:**
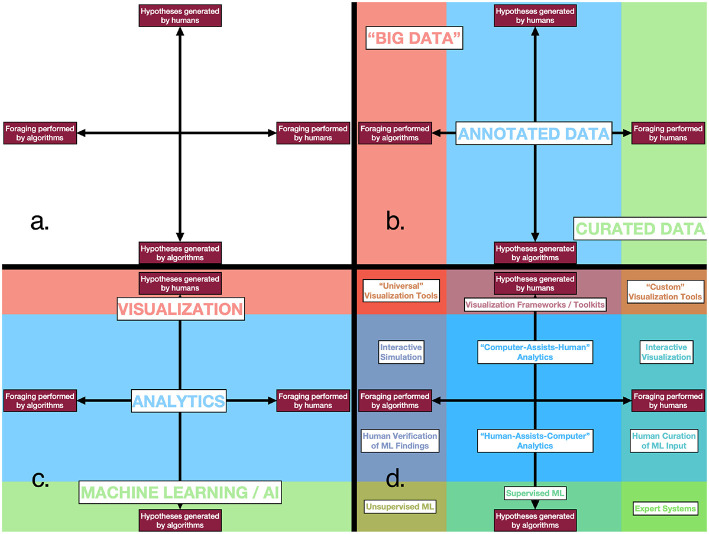
**(a)** The “space” of (immersive) analytics, inspired by the definition proposed in section 2. **(b)** One possible partitioning of the space, based on the amount of human processing involved in its collection. **(c)** Another possible partitioning of the space, based on whether the knowledge generation is being done primarily by humans or algorithms. **(d)** A partitioning of the space resulting from the combination of **(b,c)**, populated with analytic processes that illustrate the different categories.

Given that question, we propose specific research questions to support that line of inquiry:

- How can we systematically describe and classify use cases for Immersive Analytics?
  - One attempt at such a taxonomy appears in Figure 2. This taxonomy is based on the definition of immersive analytics put forward in section 2; a different definition would likely result in a different taxonomy. The space we choose to partition is the space of computer-aided analytical reasoning; the dimensions are foraging (from entirely computer-driven foraging to entirely human-driven foraging) and hypothesis generation (from entirely computer-driven to entirely human-driven). Notably, this partitioning does not specifically take into account immersion, and as such, applies to visual analytics more broadly. (One could consider the immersion of data presentation as a third dimension).
- Given such a taxonomy, can it be productively applied to the existing literature? Some research questions that might follow are: Are there categories that are over- (or under-) represented? Are there categories which are particularly effective for specific problem domains?
- One of the questions facing any analytics application is how to distribute the subtasks of analysis among machine(s) and their human user(s). Can an effective partitioning be predicted based on characteristics of the specific task or dataset? For example, can cognitive task analysis be productively applied to distribute those tasks?
- Can machine algorithms learn useful behavior from observing human user interactions with the system? Can they learn semantic relationships (e.g., spatial grouping)? Can this be enhanced by enabling more immersive interactions (e.g., positional and/or eye tracking)? It would seem that the more user interactions that can be captured, the more that could be learned from them.

### 3.2. Evaluating the Utility of Immersion

Beyond an appropriate combination of human and machine intelligences, by our definition, immersive analytics involves the use of immersive technologies. This naturally leads to the question of benefits of immersion: *Why should immersive technologies be used for visual analytics, and in what ways might they be superior to or preferred over other systems?*

In prior work, Bowman and McMahan (2007) have argued that immersion is not an all-or-nothing concept—that is, there is no sharp distinction between “immersive” and “non-immersive,” but rather a continuum of levels of immersion. In addition, immersion is multi-dimensional—many objective characteristics of a system contribute to an increased level of immersion. We have divided these into factors related to the fidelity of displays (e.g., visual field of view, haptic resolution), the fidelity of interaction (e.g., biomechanical similarity of the interaction technique to the corresponding real-world action), and the fidelity of the scenario (e.g., the realism of the physics engine's output).

With these concepts in mind, several key questions can be asked about immersive analytics:

- When does increased immersion lead to greater effectiveness, where effectiveness can be defined in terms of measurable user experience outcomes like task performance, learning, engagement, adoption, or satisfaction? Which components of immersion are key predictors of effectiveness?
- For a given task, how much immersion is needed to realize measurable benefits over traditional systems?
- How are the effects of immersion (and its components) moderated or mediated by the analysis task, the characteristics of the user, or the characteristics of the dataset?

In addition, the concept of immersion is related to many other important research questions:

- What effect does multi-modal output have? Can other (non-visual) sensory modalities be effectively used to “display” additional channels of information? Alternatively, can other (non-visual) channels be productively used to redundantly code information, enabling, for example, improved situational awareness? Are there other techniques for multi-modal data display that have not yet been considered? Can limitations in the fidelity of one display modality be compensated by another?
- What sorts of immersive technologies are most appropriate/effective for immersive analytics? For example, is AR preferred to VR? Are head-worn or world-fixed displays superior? Do we need room-scale tracking, or is it sufficient to work in a desk-scale space?
- Does “space to think” (Andrews et al., 2010) still work in immersive virtual environments? In other words, does increased virtual space—provided by large-scale tracking, high-resolution displays, and usable spatial interaction techniques—enable analysts to externalize their cognitive process in useful ways? See section 4.2 for more detailed discussion of this idea.
- In what ways do “natural” interaction techniques (those most similar to real-world interaction) contribute to improved effectiveness? Is cognitive load reduced by using such techniques?

### 3.3. Designing Immersive Analytics Systems

Section 3.2 considered immersion largely at the level of technology choice. For example, does a display with a greater field of regard result in improved outcomes? Here we consider how more immersive technologies affect the design of analytics applications. For example, given a display with a large field of regard, how should interfaces and interactions be designed to maximize its utility? Or, more broadly: *How should we design immersive user interfaces (UIs) and user experience (UX) to maximize the potential of immersive analytics?*

Some analytics applications require the integration of multiple types of data in the same workspace to be maximally productive. For the case of intelligence analysis, consider text documents, building plans (whether maps or 3D models), still photographs, security cam footage, recordings of telephone conversations—all of which might be presented as geolocated data. Questions central to this research agenda include:

- How can we define and determine appropriate spatial metaphors for different types of data?
  − How can all this data be organized so as to be “readily at hand?”
  − Can characteristics of the data be effectively mapped onto characteristics of the environment itself?
  − How best to support spatial references for users, so they don't “get lost” in the space?
- Which components of immersion can best be leveraged for analytics tasks? Some investigations along these lines include Laha et al. (2012) (head tracking, field of regard, and stereoscopic rendering), Bacim et al. (2013) (stereoscopy, head-based rendering, and display area), Laha et al. (2014) (field of regard, stereoscopy, and head tracking), and Iyer et al. (2017) (stereoscopy and field of regard).
- Given knowledge about human perception and ergonomics, how can we improve data representation and composition in immersive analytic environments? Some relevant work can be found in Bowman et al. (2003), Polys and Bowman (2004), Polys et al. (2007), and Polys et al. (2011).
  − How should one present documents/data suggested by machine intelligence (rather than resulting from user action) into the user's workspace? Should there be a distinction?
  − How can we design selection and manipulation techniques to work within and across multiple media types inside a virtual environment?
- What are appropriate travel and navigation techniques for abstract data spaces?
  − If one assumes that at least some analysts will be using immersive technologies in “deskVR” configurations (discussed in Zielasko et al., 2017), what are appropriate travel/navigation techniques that require minimal or no additional tracking/user exertion?
  − Alternatively, if one assumes that at least some analysts will have access to tracked spaces, is real walking still the best travel technique for abstract spaces? Do its benefits outweigh its drawbacks?
- What new collaboration models are enabled and/or required by the use of immersive technologies?
  − Should computational models and algorithms be represented to the user as assistants/partners/collaborators, as tools, or as something else entirely? (Consider Polys et al., 2015).
  − Is there any benefit to having them “reside” in the same space as the user?
  − Should the human user(s) explicitly interact with machine intelligence? What modalities of interaction are most natural and effective (e.g., voice, gestures, text)?
  − Furthermore, is there any benefit to having machine intelligence be represented by social actors? What effects will avatar representation, character, and trust have on the analytic outcome?
  − How does immersion better enable interaction with machine assistance/intelligence (semantic interaction)?
  − How best to notify the user that the machine has provided new information?

### 3.4. Facilitating Collaboration Through Immersion

Much of the preceding discussion has focussed on the potential benefits of immersion for a single user of an analytics system. However, many analysis tasks involve—or could potentially involve—multiple users. In Chandler et al's description of immersive analytics, presented at the start of section 2, they specifically chose to mention as an aim of such technologies “support[ing] collaboration.” Following their example, we ask: *How can immersive technologies facilitate new or improved modes of collaboration?*

- Certainly when compared to a typical workstation, physically large displays such as the CAVE and CAVE2 can better support multiple simultaneous users. However, such displays are generally only capable of generating perspectively correct imagery for a single user. Some early results from Cordeil et al. (2017b) suggest that networked HWDs—which do not share this limitation—may enable equally effective collaboration, at least under some circumstances.
  − How should user interfaces be designed to display the needed information in multiple-HWD collaborative scenarios? In Cordeil et al., they visualized each user's view frustum and fingertip in the other user's display. Is this sufficient for other tasks?
  − Cordeil et al. explored a two-user collaborative scenario in which users were tasked with evaluating network connectivity. Do the techniques they describe generalize to more users or to other types of collaborative work?
- How can immersive technologies better enable physically distributed collaboration? For example, it seems that a system that was able to track and log the focus of a user's attention and their interactions with a virtual environment could reproduce many of the benefits of colocated collaboration for users that are physically distributed but working in the same virtual space.
- Similarly, can immersive technologies better enable temporally distributed (asynchronous) collaboration? If one can track, log, and transmit information about a user's attention and actions, it seems that one would also be able to *replay* that data.
- How can we enable collaboration among devices with heterogeneous capabilities? For example, immersive devices may include large, installation-type devices such as the CAVE and CAVE2, single-user VR HWDs connected to powerful PCS such as the HTC Vive and Oculus Rift, and single-user standalone HWDs such as the Oculus Quest. While we would consider all of these to be “immersive,” they have widely varying capabilities.
  − Are there applications for which heterogeneous collaboration is advantageous? Should different user roles be mapped onto devices with different capabilities? Consider the possibility of an immersive analytics “operating theater,” where novice analysts can watch how an expert interacts with the data.

### 3.5. Changing the Analytical Process With Immersion

Highly immersive systems (which employ technologies such as those listed in section 2) enable the presentation of and interaction with information in ways that have never before been possible. It stands to reason, then, that we should carefully consider the new ways that users might be affected by the use of such systems. For example, Milk (2015) referred to virtual reality as “the ultimate empathy machine.” While one can rightly regard this claim with skepticism, it is clear that immersive analytics offer a user experience that is substantially different from that of traditional analytics systems. Therefore, one might ask: *How might the use of immersive analytics systems (as opposed to other media) affect the procedures, presentations, or products of analysis itself?* There are at least two fertile areas for investigation:

- What are the effects of immersion on the analytic process?
  − How is decision making affected by the medium of presentation or the design of the immersive representation? (see Tversky and Kahneman, 1974; Evans, 1989; Evans and Stanovich, 2013).
  − Is presentation/dissemination in immersive displays “more powerful” than traditional methods? If so, what are the associated practical or ethical concerns? (see Polys et al., 2017, 2018).
  − Is it appropriate to use VR as an “empathy machine” in the context of immersive analytics?
- How can immersive analytics be used to improve rigor and rationality of analysis?
  − How can we incorporate formal methods into immersive analytics interfaces? For example, can we explicitly require analysis of competing hypotheses, as described in Heuer (1999)?
  − Can immersive analytics provide methods and training that improve critical thinking skills?
  − How do we design immersive analytics systems that help to identify and mitigate bias and prejudice in analysis and decision-making?
  − Can immersive analytics be used to generate a verifiable (or at least externally reviewable) trace of an analyst's thought processes?
  − Can intermediate products of analysis be meaningfully preserved and annotated for educational/training/museum applications?

## 4. Immersive Analytics Research: Applications and Examples

In the previous sections, we have proposed a definition for immersive analytics and set out a research agenda for this field. In this section, we hope to make concrete the value of this research agenda by exploring how it applies to applications from the past, present, and future of immersive analytics. We begin by setting out three examples of such applications: Information-Rich Virtual Environments, Immersive Space to Think, and Immersive Archaeology. We continue by exploring how these examples have generated results that inform this research agenda, or highlighting how these projects will seek to answer questions proposed in the research agenda.

Note that we would consider all of these examples of “computer-assists-human analytics” (Figure 2D, above center). This is not entirely unreasonable, given both our research expertise and the current focus of the immersive analytics literature, which is heavily informed by visualization. Nevertheless, the class of “human-assists-computer analytics” is likely to only grow in prevalence and importance going forward, and we consider this a very important area for research in this field.

### 4.1. Immersive Analytics and Information-Rich Virtual Environments (IRVEs)

An essential property of immersive analytics is that *information and interactions are spatialized in 3D*—they exist inside a live, interactive 3D environment. Like our “old-school” paper file cabinets or piled desk, or the data mountains of Robertson et al. (1998), we use space to store our data and organize our cognition. Since the oral tradition began, spatialization has aided human memory and recall. This also seems to be the case in virtual reality, as observed by Ragan et al. (2012) and Mann et al. (2017).

So we return to our research agenda asking, “How do we spatialize information to improve the design of immersive analytics systems?” (section 3.3) One possible combination of visualization and virtual reality research is Information-Rich Virtual Environments (IRVEs), which studies how perceptual environments are augmented, or enhanced, with abstract information, including text, visualizations, and multimedia. The nuances of the problem were originally identified by Bowman et al. (1999) and Bowman et al. (2003) also articulated a research agenda that identified IRVEs as fruitful direction of study. Chen et al. (2004) examined text labels specifically. As the variety of cases began to multiply, a typology of IRVE presentation was proposed in Polys (2006) and Polys et al. (2011) that investigated the tradeoff between association and occlusion across different layout techniques.

**Figure 3:**
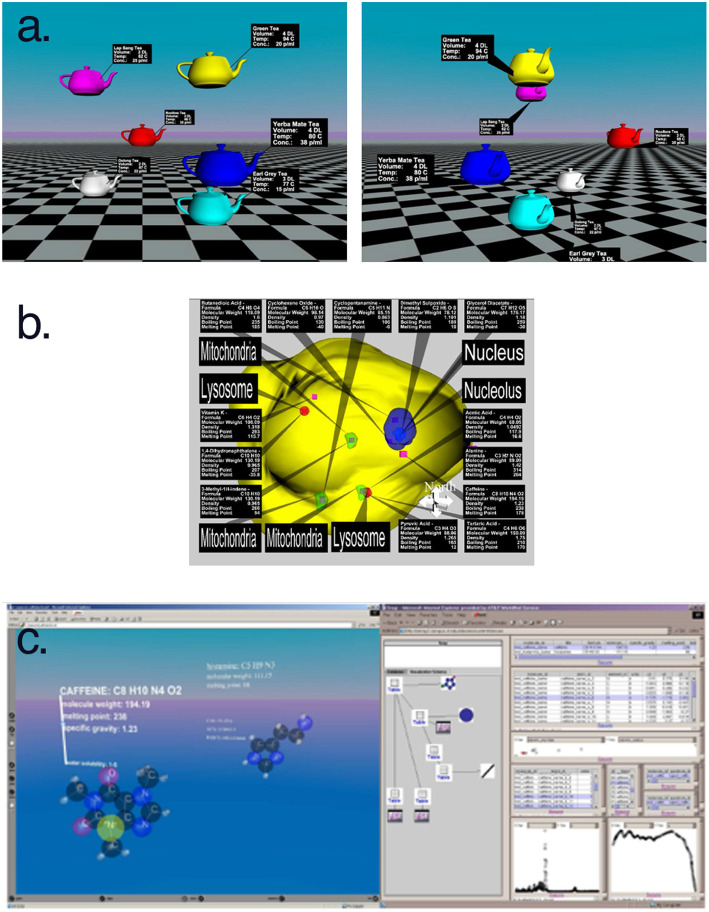
IRVE layout spaces provide support for immersive analytics through displays and interactions in **(a)** object space, **(b)** viewport space, and **(c)** display space.

Thus, IRVEs are a kind of immersive analytics. They enable users to access and apprehend additional information about the environment and its objects. Properties and attributes can be represented in the space and interactivity can support search, comparison, and pattern recognition tasks across information types, scales, and distances. Figure 3 shows a variety of layout spaces (coordinate systems) where interactive information might live: object, viewport, and display for example. This work illustrates that we can (and perhaps should) rethink our ways of designing immersive analytics workspaces and media as we move beyond the WIMP paradigm as Behr and Reiners (2008) suggest. Indeed, many of the UI and UX research questions listed in section 3.3 above apply to the visual representations and semantic interaction in an IRVE (discussed in detail by Polys and Bowman, 2004; Bowman et al., 2006; Polys et al., 2007, 2011).

**Figure 4:**
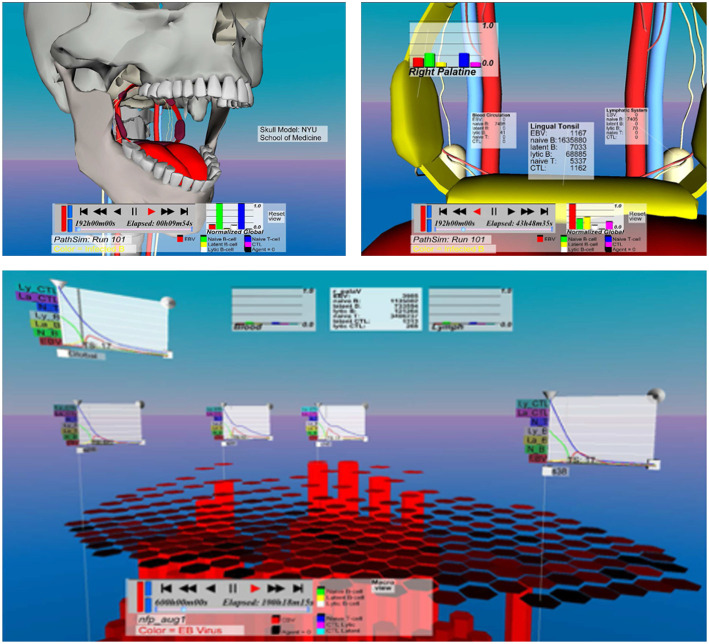
Spatialized information representations across scales in PathSim.

By spatializing visualization and interaction in a virtual environment, IRVEs support immersive analytics. The PathSim (short for Pathogen Simulation) application (described by Polys et al., 2004; Duca et al., 2007; Shapiro et al., 2008 and shown in Figure 4) is an example of how immersive analytics can be used to explore and understand the dynamics of agent-based simulations. Scientists can use PathSim to examine the types and numbers of agents (such as T-cells, B-cells, and virus particles), as well as their states and interactions across space and time. This project enabled the discovery of the fact that persistence of the Epstein-Barr virus—over 30 years—is likely due to its “hiding” in the *bloodstream*, not the lymphatic tissue.

#### 4.1.1. IRVE Research Questions

The information visualization question of “How much information can you pack into a view?” led to a related IRVE question, “How much information can be packed into an immersive display?” Early work by Ni et al. (2006) showed promising results for larger display sizes, and work by Bacim et al. (2013) showed benefits resulting from increased immersion. Polys et al. (2016) proposed an analytic model to assess the ideal capacity (bandwidth) of a display that uses visual angle and pixels per angle to put mobiles, desktops, HMDs, tiled displays, and CAVE-like displays onto the same scale. Focusing on the criterion of legibility, Iyer et al. (2017) then tested our model by investigating how text can scale between tiled HD displays and Head-Worn Displays. The analytic model based on pixels suggested a 7x advantage of a projection-based CAVE over an HMD. Experimental results revealed that for text, there was a 4x advantage. These experiments highlight our user-centered approach to quantifying the benefits of immersive analytics platforms and techniques, and as such, provide substantial insight regarding the questions raised in sections 3.2 and 3.3.

While it is clear from other VR research that immersive context can change a person's cognition—from memory (Mann et al., 2017) to spatial judgements (Laha and Bowman, 2012) to procedural training (Sowndararajan et al., 2008) to racial bias (Groom et al., 2009)—the IRVE research we describe here explores the specific contributions of pre-attentive cues (for example the depth and Gestalt cues presented by an IRVE layout) and how the user perceives these given the display. Such perceptual cues drive our pre-attentive judgements and associations between informational elements—both objects and annotations.

We advocate a deliberate approach to the design of integrated information spaces that is based on empirical evidence and driven by the goal of improving bandwidth and throughput between human and computer. There are still many open research questions for immersive analytics IRVEs. For example, we expect future IRVE experiments to explore the themes identified in section 3, such as the effective spatialization of multimedia and linked information, the effect of information layouts (perception and association) in recall and inference, and the role of display and interaction fidelity in task performance.

### 4.2. Immersive Analytics and “Space to Think”

Prior studies, including those by Andrews et al. (2011), have demonstrated the important roles that physical space plays in human knowledge generation. For example, studies of the use of large high-resolution displays to support knowledge generation from large collections of textual documents found that the physical space afforded a form of distributed cognition. Analysts naturally externalized their cognitive processes into the space, offloading cognition by spatially organizing the textual materials into visual hypothesis structures via embodied interactions, with less reliance on summarization methods such as note taking.

The ability to physically navigate the space afforded efficient information access and recall, leading to increased information synthesis. Furthermore, these behaviors were hampered when the physical navigation in a large display space was replaced by virtual navigation in a small display space (i.e., zoom and pan, controlled by a mouse). Andrews et al. (2010) referred to this phenomenon as “space to think”; this was previously discussed by Kirsh (1995), and explored further by Andrews and North (2013).

While these examples of “space to think” are 2D, one can imagine that an immersive 3D space in which to think and work might be an interesting approach to immersive analytics. In section 3.2, we asked whether the “space to think” approach still works in immersive virtual environments. This question hints at the larger idea that the existing forms of immersive analytics (immersive data visualizations and IRVEs) may not represent the only ways, or even the best ways, to take advantage of immersive technologies for data analytics.

**Figure 5:**
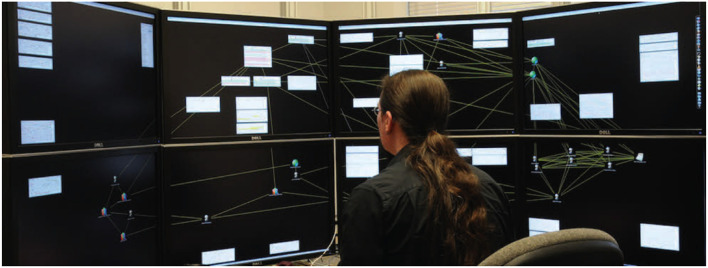
“Space to think” on a large 2D display (Andrews and North, 2012).

An important result observed by Andrews and North (2012) regarding the “space to think” phenomenon is that the human analyst's cognition becomes partially visible (Figure 5) (Earlier work by Zhang and Norman, 1994 discussed this effect in distributed cognitive tasks). That is, as the analyst organizes information in the space, it reveals clues about the synthesis activities occurring within the analyst's cognitive process. Thus, machine learning algorithms and intelligent user interfaces can be developed that observe this cognitive activity, learn from it, and respond to it to help the analyst more efficiently conduct the activity. Thus, the “space to think” becomes an efficient medium of communication between human cognition and analytical algorithm.

**Figure 6:**
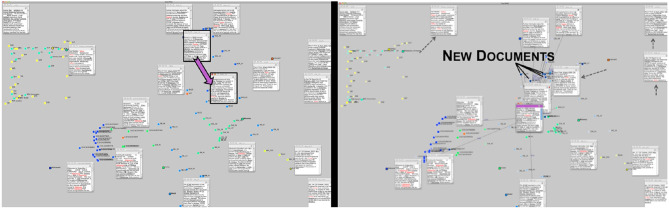
In StarSpire, a cluster created by the analyst **(Left)** is filled with more relevant documents by the system **(Right)** (Bradel et al., 2014).

Semantic interaction exploits this fact to directly support human sensemakers by recasting the analyst's interactions into analytical model parameter updates. Our work on ForceSpire and StarSpire (Figure 6) supports text analysis scenarios on large displays: as users organize documents in the space, the system learns a term importance model, enabling algorithms to forage for additional relevant documents and synthesize them on the screen within the user's organized structure. We refer to this as “synthesis driven foraging.” The system learns from the user's activity in document positioning, highlighting, annotating, and searching. This approach shields the analysts from the usability problems associated with directly manipulating algorithmic models, such as the problem of premature formality. It exploits interactions that the analyst would perform anyway, even without the presence of the algorithms, to help the analyst's knowledge generation. As a result, human and machine learn together, supporting the process of incremental formalism in knowledge generation. Results from a series of studies showed that the user and system reached a common mental/machine model, and that algorithmic support freed up the analyst to focus on higher-level knowledge generating activity (Endert et al., 2012a,b, 2013; Bradel et al., 2014).

**Figure 7:**
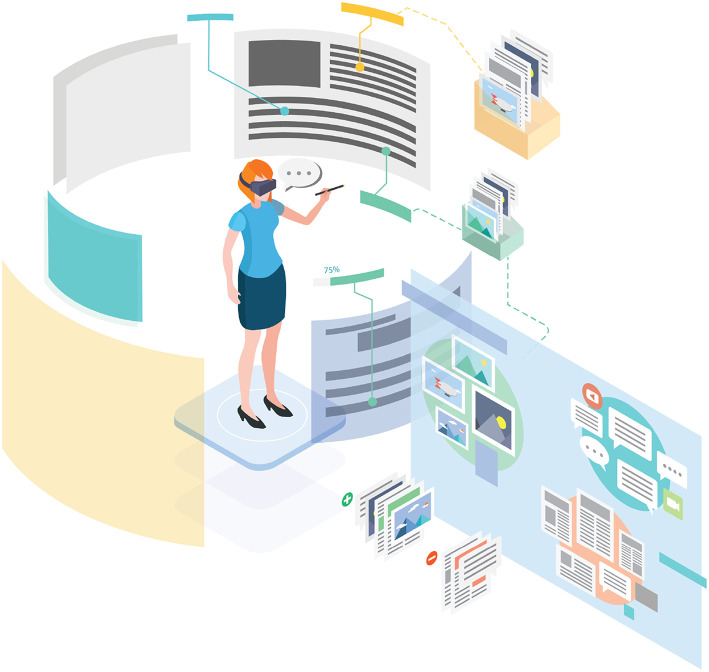
Conceptual illustration of Immersive Space to Think (IST). A human analyst uses immersive space to organize her thinking about a complex set of documents and data. The system observes interaction to recognize clusters created by the user, understand what is relevant to the user, and infer the user's hypotheses. Based on these semantic interactions, underlying machine intelligence algorithms suggest new data to consider, summarize data, and label data (e.g., with estimated credibility).

**Figure 8:**
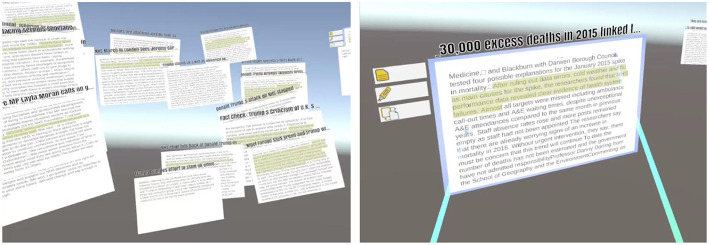
Screenshots of a prototype IST application that allows users to organize and annotate documents and regions of space in immersive VR.

In our current work, we hypothesize that immersive VR can be used to provide a more expressive, expansive space to think during analytic synthesis, and that immersive systems offer new opportunities for semantic interaction to guide machine learning algorithms and improve analytic outcomes. We call this approach Immersive Space to Think (IST). A conceptual illustration of IST is shown in Figure 7, and an early prototype is shown in Figure 8.

#### 4.2.1. IST Research Questions

Through the development and evaluation of IST, we hope to contribute knowledge that is relevant to our broader research agenda. The research plan for IST substantially overlaps with all four research areas described in section 3. As mentioned above, work by Endert et al. (2012b) suggests that the rich user interaction data available in immersive systems can be used to drive algorithmic analysis in a richer way than non-immersive input an; we intend to investigate how these data can be used to enhance machine understanding of human analytic processes. Meanwhile, the primary goal of these project is to investigate whether the “space to think” concept of Andrews et al. (2010) still applies in immersive workspaces; we also hope to generate results regarding whether increased immersion leads to greater effectiveness more generally, and how these effects might be moderated by the specific characteristics of the task, user, and data. Other questions from the research agenda that might be informed by this research project include: What are the appropriate spatial metaphors for different types of data? (section 3.3). How can immersive analytics be used to improve the rigor and rationality of analysis? (section 3.5).

### 4.3. Immersive Analytics for Archaeology

Archaeology is sometimes called the “science of destruction,” because through the very process of excavation, dig sites are disturbed to the point that reconstruction is impossible. The archaeologist in the field is privileged to the moment of discovery, and although detailed documentation of the process preserves much data that can inform interpretation, the site in its original context is forever lost as a result of the excavation process. Perhaps because of this, digital documentation of excavations, in the form of 3D scanning, is becoming increasingly commonplace, and even presenting these digital dig sites using immersive technology has been explored (Acevedo et al., 2001; Benko et al., 2004; Forte and Kurillo, 2010; Petrovic et al., 2011). Despite this, “*making full use of [large digital datasets for archaeology] remains a challenge*” [emphasis added] (Petrovic et al., 2011).

We believe that one of the reasons for this has been the unavailability of suitable hardware. As these datasets are generally 3D, large, and detailed, they are perhaps ideally suited for analysis using immersive display and interaction technologies, such as VR. With the advent of high-quality consumer-level hardware such as the HTC Vive and Oculus series of head-worn displays, this may no longer be a significant barrier.

So on the one hand, both technology improvements and updated work practices are combining to drive a “data avalanche” Petrovic et al. (2011) of high-quality 3D datasets. On the other hand, technology improvements and market pressures are combining to drive a rapid increase in quality and availability (with a simultaneous decrease in cost) of immersive displays. It seems to us that there is a natural symbiosis here, such that immersive VR is uniquely well-suited to serve the needs of domain scientists in archaeology.

That said, much archaeological evidence remains 2D, and often analog: Photographs, Harris matrices, sketches, field notes, etc. There is reason to believe that situating these data in the spatial context of a 3D reconstruction of the dig site can be a significant aid to analysis: Post-excavation analysis, typically the most time-consuming phase of the archaeology process, can consume many months and sometimes years of work, as discussed by Forte et al. (2012), but the system described by Benko, Ishak, and Feiner facilitated certain analyses “in seconds, when normally [they] can take hours to complete” (Benko et al., 2004).

We envision several ways that immersive analytics research could actually change the analytical process for archaeologists (section 3.5). First, 3D scans and models can be experienced at their true size, with a “God's-eye” view, or with a zoomed-in detail view; the relationships of rooms and artifacts and models can become an embodied perception. Also analytics can be situated in the present time where augmented or virtual reality technology could overlay historical findings on the landscape as it appears today. This would enable researchers to revisit past excavations and data at the human scale with the benefit of new knowledge and new technology. Another research thrust in immersive analytics for archaeology would be to integrate search and/or machine assistance tools, so that researchers could access external data from within the immersive analysis environment. For example, an archaeologist discovers a sword that they believe to be evidence that a site traded with a particular culture. They could then search for—or have automatically provided—other examples of swords from that culture, in order to test their hypothesis.

**Figure 9:**
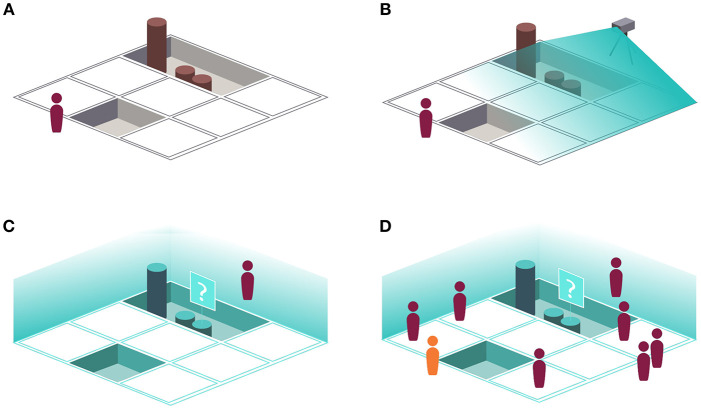
**(A)** Conventional archaeological fieldwork, with an archaeologist physically present at the excavation site. **(B)** Conventional fieldwork, with the addition of 3D scanning (LIDAR, photogrammetry, etc.) to document the ongoing excavation. **(C)** An archaeologist viewing an immersive virtual reconstruction of the excavation at a particular stage, made possible via the 3D scanning illustrated in **(B)**. Note that the system is presenting additional information about a particular artifact, possibly including high-resolution photographs, 3D scans, field notes, corroborating evidence from the Internet, etc. **(D)** Immersive archaeology applied to an educational setting.

Figure 9 presents several stages of the proposed work process, culminating in a shared IVE used for education. The figures also illustrate the machine intelligence software that can be used to view additional details and corroborating evidence regarding some aspect of the dig. Providing that structure data is captured in the field (via laser scan or photogrammetry) at a typical dig site (Figures 9A,B), the opportunity exists for rapid processing such that field scientists could review daily point cloud records with a scientist in the lab or otherwise off-site (Figure 9C). The product of this work would then be made available as an immersive learning environment for varying levels of students as well as informal learning in museums (Figure 9D).

#### 4.3.1. Immersive Archaeology Research Questions

Immersive Archaeology has the potential to change the way archaeology research is conducted, or, at least, to change the way archaeological data is recorded and reported. Making effective use of existing and yet-to-be-captured spatial and temporal datasets, though, presents a significant UI/UX design challenge; new interaction techniques and interfaces will need to be developed, which will certainly inform and be informed by the research questions proposed in section 3.3. Observations from the development and use of immersive analytical tools for archaeological research will certainly inform the questions in section 3.5; we will have the opportunity to observe in real time the impact of immersive analytics tools on a field of research.

One specific question that will Immersive Archaeology will have to address is whether and how to visualize data uncertainty. Digital representations of archaeological sites and artifacts, while constantly improving, inherently introduce some measurement uncertainty. Concern over the presentation of such data is not new, and was formally addressed in the London Charter for the Computer-based Visualization of Cultural Heritage (Denard, 2012). It will also be important to quickly determine the provenance of any given digital object. For example, a scene may contain data that is directly captured from 3D scanning, but also artist's renditions of what the environment *may* have looked like, or objects that are based on real scans, but from a different physical location or time. All of these data sources have their utility in reconstructions of historical sites, but it will be necessary to readily distinguish one from another.

## 5. Conclusion

In this article, we have explored and defined the research area posed by “immersive analytics.” We defined immersive analytics as the science of analytical reasoning facilitated by immersive human-computer interfaces. A critical result of our exploration is a further elucidation of these terms in order that we may more meaningfully discuss what does (and what does not) fall into the realm of immersive analytics. We presented and discussed several research thrusts that are implied by this definition, and posed a set of specific research questions for the immersive analytics community. We also discussed three applications (from the 1990s to ongoing research) that we believe fall under the heading of immersive analytics research, illustrating the breadth of the area. In doing so, we hope to provide both clarity and inspiration to the immersive analytics research community.

While we have briefly discussed a number of immersive system characteristics, including the list presented in section 2, some immersive systems are more promising and relevant than others. Today, we are seeing a rapid increase in quality and availability, with a corresponding decrease in cost, for six-degree-of-freedom (6-DOF) head-worn displays. Devices including the Oculus Rift, Rift S, and Quest, as well as the HTC Vive and Vive Pro, are making highly immersive technology available to the mass market. There are some differences among these devices, but in general, their immersion profiles are the very similar: Head-worn, with stereo imagery provided via one display per eye, and two hand-held controllers/wands, all of which have 6-DOF tracking. This platform has notable strengths (stereoscopy, full-field of regard, 6-DOF tracked viewpoint and hand positions) as well as weaknesses (limited display resolution, limited capacity for symbolic input, limited capacity for local collaboration). This will likely be the immersive platform of choice for the near future, as most users do not have the space or cost budget for installation-scale immersive systems such as the CAVE (Cruz-Neira et al., 1993) or CAVE2 (Febretti et al., 2013). Research regarding the immersive characteristics of such head-worn systems is likely to pay immediate dividends.

The opportunities and challenges outlined in this paper will keep researchers and practitioners busy for years to come. As new display and interaction modalities evolve, some questions will change or perhaps become more interesting. Still, we believe that the essential human-computer interaction issues we enumerate here for immersive analytics (the interactive partnership between human and computer intelligence, the value of immersive technology for new insights and productivity, designing the immersive analytic user experience, augmenting cognition to combat bias) will be the basis for research that could profoundly impact the way we as humans reason about complex questions.

## Author Contributions

RS, NP, JO, CN, and DB contributed to the conception and discussion of this research project and participated in the discussions that ultimately led to the creation of this manuscript. NP and DB performed or supervised the original research regarding IRVEs. CN and DB performed or supervised the original research regarding Immersive Space to Think. RS and JO developed the research plan regarding Immersive Archaeology.

### Conflict of Interest Statement

The authors declare that the research was conducted in the absence of any commercial or financial relationships that could be construed as a potential conflict of interest.
